# Content and activity of human liver microsomal protein and prediction of individual hepatic clearance *in vivo*

**DOI:** 10.1038/srep17671

**Published:** 2015-12-04

**Authors:** Haifeng Zhang, Na Gao, Xin Tian, Tingting Liu, Yan Fang, Jun Zhou, Qiang Wen, Binbin Xu, Bing Qi, Jie Gao, Hongmeng Li, Linjing Jia, Hailing Qiao

**Affiliations:** 1 Institute of Clinical Pharmacology, Zhengzhou University, Zhengzhou, China

## Abstract

The lack of information concerning individual variation in content and activity of human liver microsomal protein is one of the most important obstacles for designing personalized medicines. We demonstrated that the mean value of microsomal protein per gram of liver (MPPGL) was 39.46 mg/g in 128 human livers and up to 19-fold individual variations existed. Meanwhile, the metabolic activities of 10 cytochrome P450 (CYPs) were detected in microsomes and liver tissues, respectively, which showed huge individual variations (200-fold). Compared with microsomes, the activities of liver tissues were much suitable to express the individual variations of CYP activities. Furthermore, individual variations in the *in vivo* clearance of tolbutamide were successfully predicted with the individual parameter values. In conclusion, we offer the values for MPPGL contents in normal liver tissues and build a new method to assess the *in vitro* CYP activities. In addition, large individual variations exist in predicted hepatic clearance of tolbutamide. These findings provide important physiological parameters for physiologically-based pharmacokinetics models and thus, establish a solid foundation for future development of personalized medicines.

The liver is the main site of metabolic clearance in humans and is most often the focus of drug optimization and safety studies^1^. The cytochrome P450 (CYP) superfamily consists of 57 genes and constitutes the major enzyme system responsible for the metabolism of a diverse array of endogenous and exogenous compounds^2^,^3^. As a kind of membrane bound enzymes in eukaryotic cells, most of the CYPs, particularly those involved in the metabolism of drugs and xenobiotics, are located on the cytoplasmic side of the endoplasmic reticulum. With the development of tissue homogenate techniques and differential centrifugation methods, microsomal vesicles derived from the endoplasmic reticulum could be separated and subsequent evaluation of CYP enzymes showed their localization to microsomes^4^,^5^,^6^. Because of their reproducible nature, capacity for long-term storage, and extensive characterization of optimal incubation conditions, human liver microsomes (HLM) have become the dominant system used to characterize drug metabolism *in vitro*.

The amount of microsomal protein per gram of liver (MPPGL) is a critical scaling factor used in physiologically-based pharmacokinetics (PBPK) models to extrapolate *in vitro* rates of metabolism to drug clearance *in vivo*^7^,^8^,^9^. In order to assure the accuracy of predicted values for *in vivo* clearance, the MPPGL level should be determined precisely and individual variations in MPPGL should be considered. Unfortunately, to date there are only a limited number of studies concerning MPPGL amounts in human samples. Early estimates of MPPGL were limited because they were generated either from unmatched homogenate and microsomal samples^10^, or mean values of CYP content or cytochrome P450 oxidoreductase (POR) activity in homogenates and microsomes^11^. Several studies also used only a small number of livers^12^,^13^,^14^,^15^,^16^ and the background information for the samples was often incomplete. Moreover, the values for MPPGL reported by different groups used different tissue sources, different correction methods to account for losses of microsomal protein and relatively small sample sizes that in turn provided varying mean values^16^. These studies also did not give significant attention to the potential effects of individual variations in the MPPGL. Therefore, determination of the contents and individual variations in MPPGL over a large number of samples is needed in order to provide reliable physiological parameters for *in vivo* and *in vitro* research.

Traditionally, the *in vitro* metabolic activity of CYP is determined on the basis of per mg of microsomal protein (V_M_), not on per gram of liver (V_L_). However, the ultimate application of the *in vitro* metabolic study is within human tissue, the ability to obtain close estimates of *in vivo* behavior from the *in vitro* data is an important opportunity to be fully exploited. Though MPPGL were determined in relatively small size of liver tissues, considerable individual variations were found in MPPGL contents^15^,^17^. Compared with CYP activity based on liver tissue, V_M_ has obvious disadvantage for the individual variation of activity might be underestimated, because the ignored MPPGL has larger individual variations. Consequently, the V_L_ could be more appropriate to represent the *in vitro* metabolism of CYP and to assess the individual variation in CYP activities.

Given the potential for large individual variations in response to a given drug dose, substantial effort and expense may be expended during drug development, particularly for drugs that have narrow therapeutic windows, which can frequently cause severe toxicities and even death. If the safe therapeutic range of a given drug in a population can be predicted before initiating a clinical trial, the efficiency of drug development would be improved. The *in vitro*-*in vivo* extrapolation (IVIVE) method affords researchers the opportunity to produce quantitative data on drug metabolism prior to studying pharmacokinetics *in vivo*. Therefore, after the first demonstration of IVIVE in rats in 1977^18^, many subsequent efforts were concentrated on this area. However, in these early studies there was a significantly predictive bias for *in vivo* clearance from the *in vitro* metabolic data because existing variation was not considered and instead mean parameter value reconstructed from very small data sets were used^19^,^20^,^21^,^22^,^23^. Hence, further efforts were made to incorporate population variability into PBPK models to predict *in vivo* clearance^24^,^25^,^26^.

Recently, the PBPK program in the Division of Pharmacometrics at the FDA decided to bring PBPK models into the drug review process (http://www.fda.gov/aboutfda/centersoffices /officeofmedicalproductsandtobacco/cder/ucm365118.htm). However, valid predictions for *in vivo* clearance that are based on PBPK models require large numbers of different individual parameters. Although many individual characteristics can influence the outcomes of these predictions, the greatest attention has been given to variations that occur in drug metabolism, particularly that mediated by the liver^26^. The five most important parameters in predicting hepatic clearances (CL_H_) are: i) MPPGL, ii) *in vitro* metabolic clearance (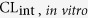), iii) liver weight (LW), iv) hepatic blood flow (Q_H_) and v) body weight (BW). Unfortunately, until now there have been no reports that used individual values for these important liver sample parameters to predict variations in *in vivo* clearance.

In order to assess the utility of individual parameters in predicting *in vivo* clearance rates, we assessed the metabolism of the sulfonylurea drug tolbutamide in liver samples. Tolbutamide is the probe of CYP2C9, which is one of the most abundant CYPs in human liver and is responsible for the metabolism of many drugs^3^. While the effects of genetic polymorphisms on CYP2C9 activities have been widely reported, experimental information demonstrating individual variations in tolbutamide metabolism *in vitro* are rather limited. An analysis of tolbutamide metabolism in HLM can be used not only to assess individual variations, but also to predict *in vivo* clearance rates. Such data will be informative for the design of personalized medicines.

In this study, 128 liver samples were collected to characterize individual variations in the contents of MPPGL and the metabolic activities of 10 CYPs based on microsomes (V_M_) and liver tissues (V_L_). For the first time, the distribution of MPPGL was assessed. The differences between V_M_ and V_L_ were compared and the correlations among V_M_ or V_L_ were analyzed. Furthermore, individual variations in the *in vivo* clearance of tolbutamide, used as a CYP2C9 probe substrate, were predicted using five important individual parameter values in large number of liver samples.

## Results

### Microsomal protein

#### Individual variation in contents

**Figure 1:**
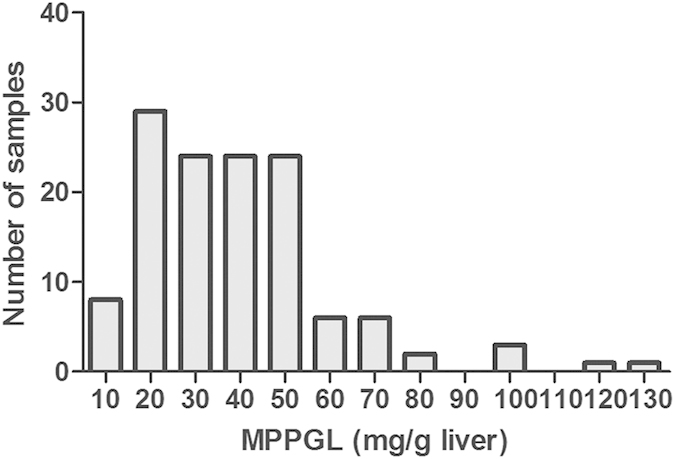
Frequency distribution of microsomal protein per gram of liver in 128 human livers.

The values for MPPGL levels in 128 samples indicated a not-normal distribution, with minimal and maximal values of 6.71 and 127.95 mg microsomal protein per gram liver, representing a 19-fold variation (Fig. 1). The mean MPPGL content ± SD was about 39.46 ± 21.57 mg/g liver. The values of MPPGL at the 2.5th and the 97.5th percentiles were 10.5 and 102.82 mg/g liver, respectively, exhibiting about a 10-fold variation. There was one extreme value (127.95 mg/g liver) and four outliers (116.50, 104.23, 97.98 and 96.97 mg/g liver) in MPPGL content. Compared with the other samples analyzed, these samples showed no extraordinary characteristics, so it can be inferred that individuals with extreme values of MPPGL do indeed exist in the population.

#### Effect of demographic factors and clinical data on contents

MPPGL content data were stratified by liver donor age, gender, smoking habit, alcohol consumption and tissue resource and then analyzed by Mann-Whitney U test or Kruskal-Wallis test. As shown in Table 1, MPPGL contents were not associated with either age (*P* > 0.05) or gender (*P* > 0.05). Smoking and alcohol consumption had no effects on MPPGL values (*P* > 0.05). We failed to detect marked disease-related differences in MPPGL contents (*P* > 0.05).

### The individual variation in metabolic activities of the CYP enzymes

#### The metabolic activities based on microsomes

**Figure 2:**
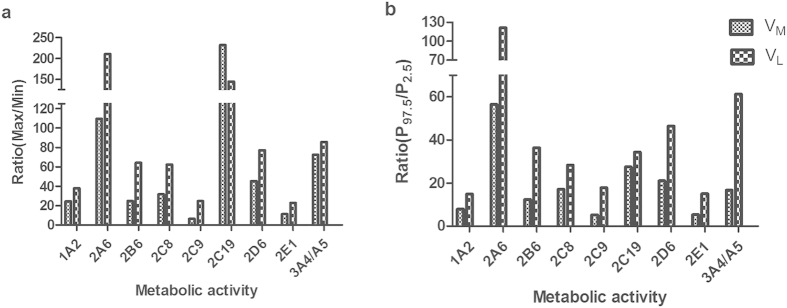
Fold-change of CYP metabolic activity based on microsomes (V_M_) or liver tissues (V_L_). The individual fold-change is expressed as the ratio between the maximal and minimal values of CYP metabolic rate (**a**) or between the 97.5th and the 2.5th percentiles of the observed CYP metabolic rate (**b**).

Metabolic rates of 10 kinds of CYPs were detected in 78 liver microsomes using probe drugs known to be specific for each enzyme. The data of CYP activities shown in Table 2 and Fig. 2 demonstrated huge individual variations. The two biggest individual variations in V_M_ took place in the activity of CYP2C19 and CYP2A6, reaching to 232 and 109-fold, followed by that of CYP3A4/5 CYP2D6, CYP2C8, CYP2B6, CYP1A2 and CYP2E1, demonstrating the fold-change of 72, 45, 31, 24, 24, and 11, respectively (Fig. 2a). Compared with other CYP members, fold-change of CYP2C9 V_M_ was much lower but still achieving 6-fold. As shown in Fig. 2b, the highest variation (57-fold) in V_M_ at 95% PI existed in the activity of CYP2A6, whereas the remaining enzymes had the following rank order: CYP2C19> CYP2D6> CYP2C8> CYP3A4/5> CYP2B6> CYP1A2> CYP2E1> CYP2C9 (28, 21, 17, 17, 12, 8, 5, and 5-fold, respectively).

#### The metabolic activities based on liver tissues

According to the contents of MPPGL determined above, individual metabolic rates per gram liver (V_M_) were obtained by multiplying the individual MPPGL values, representing the CYP activity of liver tissue (Table 2). However, when calculation of CYP activity based on liver tissue, the individual variations of CYP activities were even more pronounced (Fig. 2a). V_L_ of CYP2A6 exhibited the largest individual variation (210-fold), followed by that of CYP2C19, showing 144-fold individual variation. The remaining enzymes had the following rank order: CYP3A4/5>CYP2D6>CYP2B6>CYP2C8> CYP1A2>CYP2C9>CYP2E1 (86, 77, 64, 63, 38, 25 and 23-fold, respectively). The biggest individual variation in V_L_ at 95%PI took place in the activity of CYP2A6, reaching to 122, followed by CYP3A4/5, CYP2D6, CYP2B6, CYP2C19, CYP2C8, CYP2C9, CYP2E1, and CYP1A2, demonstrating the fold-change of 61, 46, 36, 34, 28, 18, 15 and 15, respectively (Fig. 2b).

#### The inter-individual variations in V_M_ and V_L_

As shown in Fig.2a, inter-individual variations in the V_L_ of CYPs were much higher than those of corresponding V_M_ except CYP2C19. The fold-changes in V_L_ of CYP2C9, CYP2B6, CYP2E1, CYP2C8, CYP2A6, CYP2D6, CYP1A2 and CYP3A4/5 exceeded those of corresponding CYP V_M_ by 278%, 159%, 99%, 96%, 92%, 71%, 56% and 18%, respectively, However, the fold-change in V_L_ of 2C19 decreased by 38% compared with that of CYP2C19 V_M_. When the fold-change expressed as ratio between 95% PI of the observed CYP metabolic activities (Fig. 2b), the inter-individual variation degree of V_L_ in all the CYP isoforms were higher than the corresponding V_M_. Among 10 CYPs, V_L_ of CYP2C9, CYP2B6, CYP2E1, CYP2C8, and CYP2A6 had the large amplitude of variation whereas those for CYP3A4/5 and CYP2C19 were less compared with corresponding V_M_ (Fig. 2b). Additionally, in our study the metabolic rates of CYP2C19 and CYP2A6 showed the highest fold-changes of the measured enzymes; whereas those for CYP2C9, CYP1A2 and CYP2E1 were relatively lower (Fig. 2).

#### The intra-individual variations between V_M_ and V_L_

**Figure 3:**
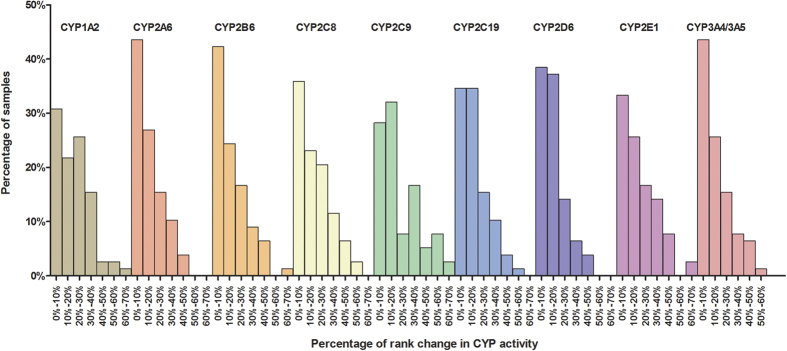
Percentage of rank change of individual V_L_ compared with corresponding V_M_ in 10 CYPs of 78 liver samples. 78 samples were ranked in ascending order according to the value of V_M_ or V_L_ for each CYP isoform, respectively. The rank change for each CYP isoform of each individual was the absolute difference value between the rank of V_M_ and the rank of corresponding V_L_. The percentage of rank change was calculated by the total samples of 78 divided by rank change absolute value of each individual and every change of 10% as a group (such as 0%-10%, 10%-20%). The percentage of the samples in each group to total samples was also calculated. Rank change of less than 10% was considered as tiny change, between 10% and 20% as moderate change, between 20% and 50% as obvious change and exceeding 50% as dramatic change.

The inter-individual variations of CYP metabolic activities in V_M_ and V_L_ were shown in Table 2 and Fig. 2. However, the intra-individual variation between V_L_ and the corresponding V_M_ in each CYP isoform of 78 samples was unknown and this situation was described by rank change percentage (Fig. 3). Rank change of individual V_L_ compared with corresponding V_M_ in 10 CYPs was obvious. For CYP1A2, CYP2C8, CYP2C9 and CYP2E1, the place change exceeding 20% accounted for 47%, 41%, 40% and 41% of the total samples, respectively, whereas those for the remaining CYPs were about 30%. Owing to the extremely high or low MPPGL amounts in some individuals, rank changes of certain CYP isoforms in these cases varied drastically. As shown in Fig. 3, the rank change of CYP2E1 in one sample experienced the most dramatic change, up 54 places (change of 69%). Next were CYP1A2 (2 cases), CYP2B6 (1 case), CYP2C9 (6 cases), CYP3A4 (1 case), CYP2C8 (2 cases), CYP2C19 (1 case), altering 41–51 places (change of 51–65%).

#### Effects of demographic factors and clinical data on CYP activities

**Figure 4:**
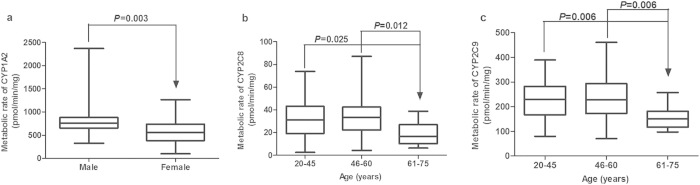
Influences of demographic factors on CYP activities. Effects of sex on the metabolic activity of CYP1A2 (**a**), effects of age on the metabolic activity of CYP2C8 (**b**) and CYP2C9 (**c**) in liver microsomes. Data are shown as box plots representing medians with minimal and maximal values.

Univariate analysis was performed to investigate whether demographic factors were associated with V_M_ and V_L_ (Fig. 4). There were no statistically significant differences (*P* > 0.05) in the metabolic activities of ten CYPs as a function of gender, age, smoking status, drinking habit or tissue resources with three exceptions. Statistically significant differences between V_M_ of CYP1A2 in male and female donors (*P* = 0.003, n = 78) were seen, which was consistent with other studies^27^,^28^. Comparing with age 3 group (61–75 years old), the V_M_ of CYP2C8 and CYP2C9 were significantly higher in age 1 (20–45 years old) and age 2 (46–60 years old) groups (Fig. 4b, c). Yang *et al.*^29^ also found age had a substantial impact on the activity of CYP2C9.

### Association between MPPGL contents and CYP activities

#### Association based on microsomes

Spearman correlation analysis was used to identify the correlations between MPPGL contents and V_M_ for 10 CYP, respectively, and the results showed there was no significant correlation between MPPGL contents and V_M_ of 10 CYPs (*P* > 0.05) (Supplementary Fig. S1).

#### Association based on liver tissues

In contrast to V_M_, there were strong correlations (*r*≥0.6, *P*<0.001) between MPPGL contents and V_L_ of CYPs besides CYP2A6, CYP3A4/5, CYP2C8 (moderate correlations, 0.3≤*r*<0.6, *P*<0.001) (Supplementary Fig. S2).

### Prediction of tolbutamide hepatic clearance

#### *In vitro* clearance of tolbutamide

The mean ± SD of the V_max_ and K_m_ for tolbutamide 4-hydroxylation was 255.82 ± 79.48 (range 83.76 to 454.80) pmol/mg/min and 235.73 ± 99.78 (range 101.20 to 531.90) μM, respectively. Both the V_max_ and K_m_ values displayed individual variations of 5-fold, with the variations at 95% percentiles interval (PI) still reaching to 5-fold. The values of CL_int, *in vitro*_ for 4-OH-tolbutamide by CYP2C9 are shown in Supplementary Table S1 and the not-normal distribution of the values displayed a 21-fold individual variation between the highest and lowest values.

All parameters used to predict the hepatic clearance of tolbutamide in the 78 HLM samples are listed in Supplementary Table S1. The variable degrees of the other three parameters were relatively lower, with only BW variations reaching 2-fold.

#### Prediction of tolbutamide hepatic clearance

The predicted and observed CL_H_ for tolbutamide are shown in Table 3. The mean values for the predicted CL_H_ of tolbutamide obtained by seven methods were all 0.113 ml/min/kg, but the predicted ranges showed obvious variations. Method G gave merely the mean value, whereas method A predicted the largest individual variation (51-fold between the highest and lowest values). Even at the 95% PI, the predicted variation still reached 30-fold with method A. Using only individual MPPGL or CL_int,*in vitro*_ values, method B and C also displayed large individual variations (about 13- and 20-fold, respectively). In contrast, the ranges of the predicted CL_H_ for tolbutamide were relatively narrow as calculated with method D, E and F, with less than 2-fold variation between the minimal and maximal values. The effects of individual values for the five parameters on the predicted CL_H_ for tolbutamide are presented in Supplementary Fig. S3. Together these results indicate that ignoring individual variations in parameter values would lead to a failure to identify individuals who fall at the extremes of the population.

#### Prediction accuracy

As shown in Table 4, the mean average fold-error (AFE) values calculated by all of the seven methods (A-G) were the same (0.58), but the variable degrees of AFE were quite different. Method G gave merely the mean value, whereas method A predicted the largest individual variation in AFE (50-fold between the highest and lowest values). Even at the 95% PI, the predicted variation still reached 30-fold with method A. Using only individual MPPGL or CL_int,*in vitro*_ values, method B and C also displayed large individual variation in AFE (about 13- and 20-fold, respectively). In contrast, the ranges in AFE values yielded by methods D, E and F were relatively narrow, with less than 2-fold variation between the minimal and maximal values. The percentage of predictions that fell within 2-fold of the *in vivo* value were different between different methods. By not incorporating the individual variation of these five parameters, or using the less variable degree in individual Q_H_ and LW, methods G, E and D provided overall accuracy. However, owing to the large individual variations seen for MPPGL and CL_int,*in vitro*_ values, only 42%, 46% and 52% of the samples were within the 2-fold error as determined by method A, B and C, respectively.

## Discussion

This is the first extensive study to investigate the distribution of microsomal protein contents in a large number of normal liver samples. The not-normally distributed MPPGL values in 128 samples varied from 6.71 to 127.95 mg/g liver and the range at 95% PI were 10.5 to 102.82 mg/g. Meanwhile, the metabolic activities of 10 CYPs were detected in microsomes and liver tissues, respectively, which showed huge individual variations and the variation for some CYPs could reach over 200-fold. Compared with microsomes, the activities of liver tissues were much suitable to express the individual variations of CYP activities. Furthermore, individual variations in *in vivo* clearance of tolbutamide were successfully predicted with the individual parameter values and we provided a valuable database of metabolic parameters for normal livers for use in PBPK modeling.

Previous studies demonstrated that the CYP content and POR activity used for correcting the loss of MPPGL in homogenates are all essentially microsomal in origin^30^, and the corrected values of MPPGL based on either CYP contents or POR activities are comparable and not significantly different^15^,^16^. The average MPPGL value of 39.46 mg/g based on the POR activity from 128 liver tissues was essentially the same as the value obtained for 38 liver samples (40 mg/g^16^) and is in good agreement with the result of Pelkonen *et al.* (36 mg/g^11^). Thus, there is general agreement between various laboratories using a variety of methodologies. While our mean_geo_ value of MPPGL is greater than the mean_geo_ value of 28 mg/g determined by Barter *et al.*^17^, it is noteworthy that this result came from unpublished data and thus detailed information for the donors was not available. In addition, two other studies reported higher average MPPGL levels, in which the value of 77 mg/g was obtained from only four liver tissue samples^12^ and the determination of 53 mg/g was achieved by ELISA^14^. The use of ELISA to correct for microsomal losses is not a common approach, and this disparity may be due to differences in the correction methods used in these studies (i.e., POR activity vs. ELISA). In a previous study by this group a mean MPPGL value of 20 mg/g^13^ was obtained by using glucose-6-phosphatase activity to correct for microsomal loss.

To date only limited laboratory measurements concerning the variations in the contents of MPPGL were available and the largest reported variation was approximately 6-fold^17^. However, in the present study up to a 19-fold variation was detected and even at the 95% PI there was still a 10-fold variation, which is much higher than that reported in the literature. It should be emphasized that the present study used a larger number of samples and the range of MPPGL values obtained here overlapped with the results reported by other investigators^16^,^17^. The determination of MPPGL is time-consuming and requires the coordination of several operators. To ensure the accuracy of the MPPGL results, the effect of inter-operator differences on MPPGL values was investigated. Pooled human livers (n = 5) were respectively assessed by three operators in triplicate and the results showed that the difference in the MPPGL values obtained by different operators was not substantial and was less than 20%, which is in good agreement with a study by Wilson *et al.*^31^. As such, the large variation in the MPPGL levels seen in our study most likely represents the true biological variability present in the population. Taken together, our study offers physiological values for MPPGL in normal liver samples that have clear background information.

In general, the *in vitro* activity of CYP is based on microsomes (V_M_) and a number of studies have reported the high degrees of individual variations in the V_M_ (such as 30-, 45-, 405-, 1790-, 30- and 124-fold for CYP2A6, CYP2B6, CYP2C19, CYP2D6, CYP2E1 and CYP3A4^28^,^32^,^33^,^34^,^35^, respectively). Our study also found large individual variations in V_M_ (Table 2 and Fig. 2). However, as the actual contents of MPPGL are unusually unknown, so far no reports are available regarding the metabolic activities of CYP on the basis of per gram liver (V_L_). Since the determination of MPPGL amount in our study, it has been found that the individual variations of CYP V_L_ were much higher than those of corresponding V_M_. As shown in Fig. 2, the degree of the overall variation of V_L_ for CYP2C9, CYP2B6, CYP2E1, CYP2C8 and CYP2A6 increased substantially comparing with those of corresponding V_M_ of CYPs. Meanwhile, the intra-individual variation between V_L_ and the corresponding V_M_ in each CYP isoform of 78 samples was large. Especially for CYP1A2, CYP2C8, CYP2C9 and CYP2E1, more than 40% of the samples changed more than 20% of the total rank and for some cases, the rank change over more than 60% (Fig. 3). The reason for large rank change is due to the variation in MPPGL, especially the effect of extreme values in MPPGL. For example, the highest MPPGL contents in some cases resulted in the huge increase of rank whereas the lowest led to the dramatic decrease of rank. The change of the rank for each individual between V_M_ and the corresponding V_L_ indicated that V_M_ didn’t equal to V_L_ especially for the individuals with drastic rank changes. Taken together, it can be concluded that V_L_ is preferred over the V_M_ in representing the individual variations of CYP metabolic activity for the latter may mask the real individual variations of enzyme activities.

There are four stages in the IVIVE strategy, in which MPPGL, CL_int, *in vitro*_, LW, Q_H_ and BW are necessary parameters^9^. These parameters thus play a vital role in the accurate prediction of *in vivo* clearance rates. However, to our knowledge the various values for these important parameters used in the IVIVE were only single mean values^7^,^36^ and no consideration was given to variations in these parameters. Here, for the first time the individual values for the five of parameters were used to predict individual variations in hepatic tolbutamide clearance in a large number of liver samples. As shown in Table 3 and Supplementary Fig. S3, seven methods were designed to predict the hepatic clearance of tolbutamide. Because the mean and individual values of the five parameters used with the seven methods came from the same population, the mean values of the tolbutamide CL_H_ predicted by each of the methods were the same. However, the variable degrees of the predicted CL_H_ were quite different. Method G, which is a rather traditional “point” to “point” predictive manner, only provided the mean value and thus could not offer information on variations in the population. Method A, as a new approach that was used in this study, incorporated the individual variations of the five parameters into the prediction and in turn displayed as much as a 51-fold individual variation in the distribution of the predicted CL_H_ for tolbutamide. Thus, the ability to predict individual variations in CL_H_ is superior to the estimate of a mere mean. For future drug research and development, the prediction of the CL_H_ range in the human body prior to the initiation of clinical studies can provide information about the effective range and toxic doses that can improve the efficiency of research on novel therapeutics. For older drugs that are currently in clinical use and have large individual variations, method A can help to re-assess the safety of these drugs and to provide a firm basis for the design of personalized treatments.

In contrast, methods B-F evaluated the effect of individual variation of a single parameter (MPPGL, CL_int, *in vitro*_, LW, Q_H_ or BW) on the prediction. Each parameter could affect the individual variation of the predicted CL_H_, but the contributions of the five parameters were different. The large individual variation predicted by method A was mainly caused by the variation in MPPGL and CL_int,*in vitro*_, so MPPGL and CL_int,*in vitro*_ thus could play essential roles in the process of IVIVE. Among all the parameters used in IVIVE, individual values for MPPGL and CL_int,*in vitro*_ were not easily obtained, therefore prediction of the individual variation in CL_H_ would be highly difficult. In this study, individual values for the five kinds of important parameters were determined and used to predict the CL_H_ of tolbutamide.

In order to assess the accuracy of the predictions, a comparison of the predicted hepatic clearance with the *in vivo* clearance in humans is needed. Due to a lack of human intravenous pharmacokinetic data in the Chinese population, data suitable for assessment of tolbutamide CL_H_ predictions in Caucasian populations were selected from four previous studies (Table 3). As was previously known, CYP2C9 is highly polymorphic and the frequencies of CYP2C9*2 and CYP2C9*3, which exhibit poor enzymatic activity compared to CYP2C9*1^37^, were higher in Caucasians compared to that in Chinese populations. Therefore, a reasonable assumption could be made that the metabolic activity of CYP2C9 should differ between Caucasian and Chinese populations. However, the CL_int, *in vitro*_ of tolbutamide in Caucasian population (mean ± SD, range: 1.0 ± 0.2, 0.5–2.5; 1.35 ± 1.23, 0.27–4.0 μl/mg/min) were reported by McGinnity *et al.*^38^ and Carlile *et al.*^39^, respectively, which were in good agreement with those of Chinese people (mean ± SD, range: 1.32 ± 0.75, 0.20–4.18 μl/mg/min, this study). The consistency of the 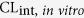 of tolbutamide in these two ethnic groups might suggest that gene polymorphisms in CYP2C9 have little effect on the metabolism of tolbutamide in ethnic Chinese and Caucasians. Consequently, the *in vivo* CL_H_ for tolbutamide derived from the Western population should be suitable for comparisons with the predicted CL_H_ for tolbutamide in Chinese patients. The predicted mean value within 2-fold of actual values showed that each of the methods was accurate in predicting the CL_H_ of tolbutamide (Table 4).

However, more than half of the samples fell outside of the 2-fold error for method A, B and C, which may explain, at least in part, the bias that exists in most IVIVE studies. Traditionally, IVIVE employed only mean values to make predictions and as such cannot provide a range of AFE. In fact, as observed in our study, large variations indeed exist in many drug metabolism steps, so a method that incorporates into the IVIVE individual variations in each step can be a suitable way to make accurate predictions for *in vivo* CL_H_.

In conclusion, MPPGL values were determined and considerable individual variations in the contents were found in Chinese population. The metabolic activity of CYP based on liver tissue is a new method to assess the *in vitro* metabolic activity of CYP and superior to the metabolic activity of CYP based on microsomes. For the first time the individual values of five different parameters were used to predict individual variations in hepatic clearance in a large number of liver samples and variations in the *in vivo* clearance rates of tolbutamide were successfully predicted. These findings provide important physiological parameters for PBPK and furthermore, build a solid foundation for future development of personalized medicines.

## Methods

### Materials and Chemicals

All probe drugs and part metabolites used in this work were purchased from the National Institute for the Food and Drug Control (China), including phenacetin, coumarin, bupropion, paclitaxel, tolbutamide, omeprazole, dextromethorphan, chlorzoxazone, midazolam and paracetamol (phenacetin metabolite). Other metabolites (7-OH-coumarin, 4-OH-bupropion, 6-OH-Paclitaxel, 4-OH-tolbutamide, 4-OH-omeprazole, 3-methoxymorphinan, 6-OH-chlorzoxazone and 1-OH-midazolam) were obtained from Toronto Research Chemicals, Inc. (Canada). Methanol and acetonitrile were HPLC grade and were purchased from Siyou Chemical Reagent Co. (China). Reduced nicotinamide adenine dinucleotide phosphate (NADPH) and horse cytochrome C were obtained from Solarbio Science and Technology co. (China).

### Human liver samples

One hundred and twenty-three Chinese liver tissues were previously collected^40^ from patients undergoing liver surgery during 2012 and 2014 in the first affiliated hospital of Zhengzhou University, the People’s Hospital of Henan Province, and the Tumors’ Hospital of Henan Province, respectively, besides 5 newly collected samples. The study was approved by the ethics committees of Zhengzhou University and written informed consent was obtained from each patient. All experiments were performed in accordance with the approved guidelines of Zhengzhou University ethics committees. Detailed information for each patient was well-documented and included gender, age, body height, body weight, smoking habits, alcohol consumption, clinical diagnosis, regular drug intake before surgery, previous history, allergic history, pathological diagnosis, imaging examination and laboratory test data (including, but not limited to, results from routine blood analysis, liver function tests and renal function tests). Liver samples from tumorous patients were 2 cm distant from the tumor tissues. Samples from normal livers were collected, with liver health confirmed by liver function tests, histopathological analysis and imaging examination by ultrasonography or CT. All liver samples were frozen immediately after removal and stored in liquid nitrogen until use.

### Preparation of liver microsomes

Tissue samples were thawed on ice and weighed. The samples were finely homogenized on ice using a glass homogenizer in 0.05 M Tris-HCl (pH 7.0) buffer containing 1.12% w/v KCl and 1.12% v/v EDTA (10 ml per gram liver). After mixing, 0.5 ml of the homogenate was retained for POR activity analysis while the remaining sample was centrifuged at 9,000 × g for 20 min at 4 °C. The supernatant was collected and centrifuged at 100,000 × g for 1 hour at 4 °C with a Beckman Optima L-100K ultracentrifuge. The resulting microsomal pellet was resuspended in 0.15 M Tris-HCl (pH 7.6) buffer and centrifuged for an additional hour at 100,000 × g at 4 °C. The final microsome pellet was suspended in 0.25 M sucrose (2 ml per gram original sample). Both the homogenate and microsomal suspension were frozen in liquid nitrogen and stored at −80 °C until analysis. Microsomal protein concentrations were determined according to the Bradford method.

### Determination of microsomal protein per gram of liver (MPPGL) levels

The activity of POR as measured in homogenates and microsomes produced from the same liver tissue sample was used to estimate the amount of MPPGL^41^. The POR assay is based on the rate of cytochrome C reduction by liver microsomes^42^. The reaction was conducted in 200 μl solution with 0.3 M potassium phosphate buffer (pH 7.7), 0.2 mM horse cytochrome C, and 5 μg microsomal proteins. Reactions were initiated by the addition of 20 μl 10 mM NADPH to 200 μl assay mixture for a total volume of 220 μl. The rate of cytochrome C reduction was determined from the rate of increase in absorbance at 550 nm produced by the reduced form of cytochrome C using a BioTek Synergy H1MD Multi-Mode microplate reader in the kinetic mode before and after the addition of NADPH (0–5 min). MPPGL values were calculated with the following equation^15^: MPPGL = {rate of reduction_homogenate_ (nM/min/g liver)}/{rate of reduction_microsome_ (nM/min/mg microsomal protein)}.

### Measurement of CYP metabolic activities

Marker activities selective for individual CYP isoforms were determined at single concentrations in individual assays by incubation of 0.2–0.5 mg microsomal protein, 1 mM NADPH and the respective substrate (400 μM phenacetin for CYP1A2, 20 μM coumarin for CYP2A6, 500 μM bupropion for CYP2B6, 40 μM paclitaxel for CYP2C8, 1500 μM tolbutamide for CYP2C9, 250 μM omeprazole for CYP2C19, 320 μM dextromethorphan for CYP2D6, 500 μM chlorzoxazone for CYP2E1 and 50 μM midazolam for CYP3A4/5). In addition, seven different concentrations of tolbutamide (31.25 to 2000 μM) were examined to determine the V_max_ and K_m_ of 4-OH-tolbutamide and the *in vitro* CL_int_ of tolbutamide was calculated using the following equation: CL_int *in vitro*_  = V_max_/K_m_. Incubation conditions were ensured linear metabolite formation with respect to reaction time and protein concentration. Each reaction was terminated after specified incubation period by adding 20 μl ice-cold acetonitrile or 1 ml ethyl acetate or perchloric acid and metabolite concentrations were determined by HPLC-UV or HPLC-FLD.

### Prediction of tolbutamide hepatic clearance

The CL_int,*in vitro*_ values obtained for tolbutamide were scaled to *in vivo* clearance by the following equations. The whole liver intrinsic clearance (CL_int, liver_) was estimated as:


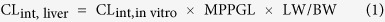


Where LW is liver weight and BW refers to body weight. According to the body weight given for each patient, the liver weight (LW) was calculated from the liver volume (LV) multiplied by liver density, where LV (ml) = 12.5 × BW (kg) + 536.4^43^ and liver density is 1.001 g/ml^44^. The formula for liver volume was derived from data collected from a Chinese population.

The hepatic clearance (CL_H_) of tolbutamide hydroxylation was then predicted using the well-stirred model:


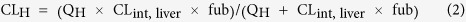


Where *f*ub is fraction unbound in blood (0.0982)^8^,^45^ and Q_H_ is liver blood flow, which is often expressed as a percentage of cardiac output because of the difficulty in determining the Q_H_. Q_H_ was assumed to be 24.5% of cardiac output^7^. The values for cardiac output originated from data for normal Han Chinese males (n = 783) and females (n = 805) and the mean values from each group were selected according to the age and gender of the donors in this study^46^.

Both the mean and individual values for the five different parameters observed in this study (MPPGL, CL_int, *in vitro*_, LW, Q_H_ and BW) were used to predict the hepatic clearance of tolbutamide. According to the different combinations of mean and individual values of the five parameters, seven methods were employed. Method A used individual values for each parameter for that particular liver (n = 78). Method B used the individual MPPGL value and the mean value of the remaining four parameters. Similar to method B, methods C, D, E and F used the individual CL_int, *in vitro*_, LW, Q_H_ or BW value, respectively, and the mean value of the remaining four parameters. Method G used the mean value of the five parameters for 78 livers.

Due to a scarcity of data for the Chinese population, observed intravenous clearance values for tolbutamide were obtained from *in vivo* studies performed with healthy Caucasian subjects.

The accuracy of the predictions was assessed from the average fold-error (AFE). A two-fold precision limit corresponds to 0.5–2 of AFE values, where AFE = 10^(∑logPredicted/Observed)/N 21^. N refers to the number of separate reports in the literature concerning tolbutamide intravenous clearance.

### Statistical Analyses

The normality of the data distribution was assessed using the method of Kolmogorov-Smirnov and Shapiro-Wilk. Because most data sets were not normally distributed, nonparametric methods were generally used for statistical analyses. The Mann-Whitney *U* test was used for pairwise comparison and the Kruskal-Wallis H test was applied for multiple pairwise comparisons. Non-parametric Spearman rank correlation analysis was performed to calculate the correlation coefficient (*r*). A *P* value < 0.05 was considered statistically significant (two-tailed). SPSS statistics 17 software was used for data management and statistical analyses. Graphs were generated using GraphPad Prism software 5.04.

## Additional Information

**How to cite this article**: Zhang, H. *et al.* Content and activity of human liver microsomal protein and prediction of individual hepatic clearance *in vivo*. *Sci. Rep.* **5**, 17671; doi: 10.1038/srep17671 (2015).

## Supplementary Material

Supplementary Information

## Figures and Tables

**Table 1:** Donor characteristics of human liver samples and MPPGL content in donor subgroups (n = 128).

Variables	Group	Number	Percentage(%)	MPPGLcontent (mg/g)(Mean ± SD)
Gender	Male	40	31.3	41.50 ± 21.21
	Female	88	68.8	38.53 ± 21.79
Age (years)	20–45	51	39.8	37.71 ± 21.31
	46–60	63	49.2	40.59 ± 22.06
	61–75	14	10.9	40.69 ± 21.45
Smoking	Non-smoking	115	89.8	39.10 ± 21.86
	Smoking	13	10.2	42.58 ± 19.30
Drinking	Non-drinking	115	89.8	39.13 ± 21.93
	Drinking	13	10.2	42.36 ± 18.64
Medical diagnosis	Hepatic cavernous hemangioma	93	72.7	38.25 ± 21.93
	Metastatic carcinoma	10	7.8	40.79 ± 19.07
	Cholelithiasis	9	7.0	41.03 ± 28.73
	Gallbladder cancer	5	3.9	50.91 ± 21.95
	Hepatic cholangiocarcinoma	7	5.5	45.42 ± 15.45
	Hepatocellular carcinoma	4	3.1	35.99 ± 12.68

**Table 2:** Metabolic activities of the individual CYP enzymes in human livers (n = 78).

	Probe drug (Metabolite)		V_M_ (pmol/min/mg protein)	V_L_ (nmol/min/g liver)
CYP1A2	phenacetin (paracetamol)	Median	677.94	22.12
		Range	97.37–2368.14	3.16–119.91
		95% PI	169.20–1344.69	4.77–71.38
CYP2A6	coumarin (7-OH-coumarin)	Median	267.92	8.19
		Range	7.20–788.56	0.22–47.08
		95% PI	12.19–689.73	0.35–42.83
CYP2B6	bupropion (4-OH-bupropion).	Median	46.67	1.68
		Range	11.75–290.89	0.28–17.63
		95% PI	12.16–151.14	0.31–11.31
CYP2C8	paclitaxel (6-OH- Paclitaxel)	Median	31.50	0.96
		Range	2.74–87.23	0.12–7.71
		95% PI	4.34–74.27	0.17–4.81
CYP2C9	tolbutamide.(4-OH-tolbutamide)	Median	222.70	7.29
		Range	70.00–461.07	1.51–37.61
		95% PI	76.17–391.49	1.96–34.92
CYP2C19	omeprazole (4-OH-omeprazole)	Median	97.62	3.64
		Range	1.40–325.03	0.14–19.58
		95% PI	9.98–274.83	0.51–17.62
CYP2D6	dextromethorphan (3-methoxymorphinan)	Median	68.16	2.11
		Range	4.93–222.34	0.19–14.49
		95% PI	9.06–190.03	0.30–14.15
CYP2E1	chlorzoxazone (6-OH-chlorzoxazone)	Median	486.92	16.03
		Range	140.81–1604.44	3.28–74.52
		95% PI	198.24–1061.97	4.60–69.28
CYP3A4/5	midazolam (1-OH-midazolam)	Median	836.56	29.05
		Range	57.24–4144.93	2.93–251.26
		95% PI	178.15–2967.83	3.55–217.79

V_M_: metabolic rate of the individual CYP enzyme based on per mg microsomal protein; V_L_: metabolic rate of the individual CYP enzyme based on per gram liver tissue; PI: percentile interval.

**Table 3:** Values for hepatic clearance of tolbutamide 4-hydroxylation and their variations.

	n	Range	Fold change (Max/Min)	Mean ± SD	95% PI	Fold change (95% PI)
Predicted CL_H_ (ml/min/kg)						
Method A	78	0.011–0.559	50.8	0.113 ± 0.095	0.012–0.365	30.4
Method B	78	0.027–0.341	12.6	0.113 ± 0.065	0.038–0.311	8.2
Method C	78	0.018–0.353	19.6	0.113 ± 0.064	0.026–0.268	10.3
Method D	78	0.093–0.142	1.5	0.113 ± 0.011	0.095–0.139	1.5
Method E	78	0.1128–0.1130	1.0	0.113 ± 0.000	0.1128–0.1130	1.0
Method F	78	0.079–0.161	2.0	0.116 ± 0.019	0.082–0.155	1.9
Method G	78	—	—	0.113	—	—
Observed CL_H_ (ml/min/kg)						
Back *et al.*^47^	7			0.260 ± 0.100		
Back *et al.*^47^	6			0.226 ± 0.024		
Back *et al.*^47^	6			0.239 ± 0.050		
Miners *et al.*^48^	6			0.171^a^		
Wilner *et al.*^49^	6			0.147 ± 0.013		
Wing *et al.*^50^	7			0.159^a^		

Method A used the individual values for each parameter (MPPGL, CL_int,_ *in vitro*, LW, Q_H_ and BW) for 78 livers. Method B used the individual MPPGL value and the mean values for the remaining four parameters. Similar to method B, method C, D, E and F used the individual CL_int, *in vitro*_, LW, Q_H_ or BW value, respectively, and the mean values for the remaining four parameters. Method G used the mean value of the five parameters for that particular liver.

^a^No SD is available. PI: percentile interval.

**Table 4:** Comparing the accuracy of predictions using different methods.

Method	AFE			
	Mean ± SD	Range	95% PI	% within of 2-fold error
A	0.58 ± 0.48	0.057–2.86	0.062–1.86	42
B	0.58 ± 0.33	0.14–1.74	0.20–1.59	46
C	0.58 ± 0.33	0.09–1.80	0.13–1.37	54
D	0.58 ± 0.06	0.47–0.72	0.48–0.71	92
E	0.58 ± 0.00	0.577–0.578	0.5767–0.5776	100
F	0.59 ± 0.10	0.40–0.82	0.42–0.79	82
G	0.58	−	−	100

AFE (average fold-error) was used to assess the accuracy of predictions while a two-fold precision limit corresponds to 0.5–2 of AFE values. Method A used individual values for each parameter (MPPGL, CL_int, *in vitro*_, LW, Q_H_ and BW) for 78 livers. Method B considered the individual MPPGL value and the mean values for the remaining four parameters. Similar to method B, methods C, D, E and F used the individual CL_int, *in vitro*_, LW, Q_H_ or BW value, respectively, and the mean value for the remaining four parameters. Method G used mean values for the five parameters for that particular liver. PI: percentile interval.
