# Supplementary material for: Content and activity of human liver microsomal protein and prediction of individual hepatic clearance in vivo
Source: Sci Rep. 2015 Dec 4;5:17671. doi: 10.1038/srep17671 (PMC4669488; doi:10.1038/srep17671)
Supplement: Supplementary Information [file srep17671-s1.pdf]

Supporting information

Content and activity of human liver microsomal protein and prediction of individual hepatic clearance in vivo

Haifeng Zhang [#], Na Gao [#], Xin Tian, Tingting Liu, Yan Fang, Jun Zhou, Qiang Wen, Binbin Xu, Bing Qi, Jie Gao, Hongmeng Li, Linjing Jia and Hailing Qiao *

Institute of Clinical Pharmacology, Zhengzhou University, Zhengzhou, China

Supplementary Table S1. Values for five parameters used to predict the hepatic tolbutamide clearance (n = 78)

	Range	Mean ± SD	95% PI
MPPGL (mg/g)	9.93-127.95	41.93 ± 24.50	14.23-116.79
$CL_{int,in\ vitro}$ (μl/mg/min)	0.20-4.18	1.32 ± 0.75	0.30-3.16
Liver Weight (g)	1100.00-1688.09	1341.67 ± 136.96	1124.40-1651.49
Q_H (ml/min)	1205.40-1629.25	1322.71 ± 103.02	1205.40-1629.25
Body Weight (kg)	45-92	64.31 ± 10.95	46.95-89.08

MPPGL: microsomal protein per gram of liver; $CL_{int,\ in\ vitro}$: in vitro metabolic clearance; Q_H: hepatic blood flow.

Supplementary Figures

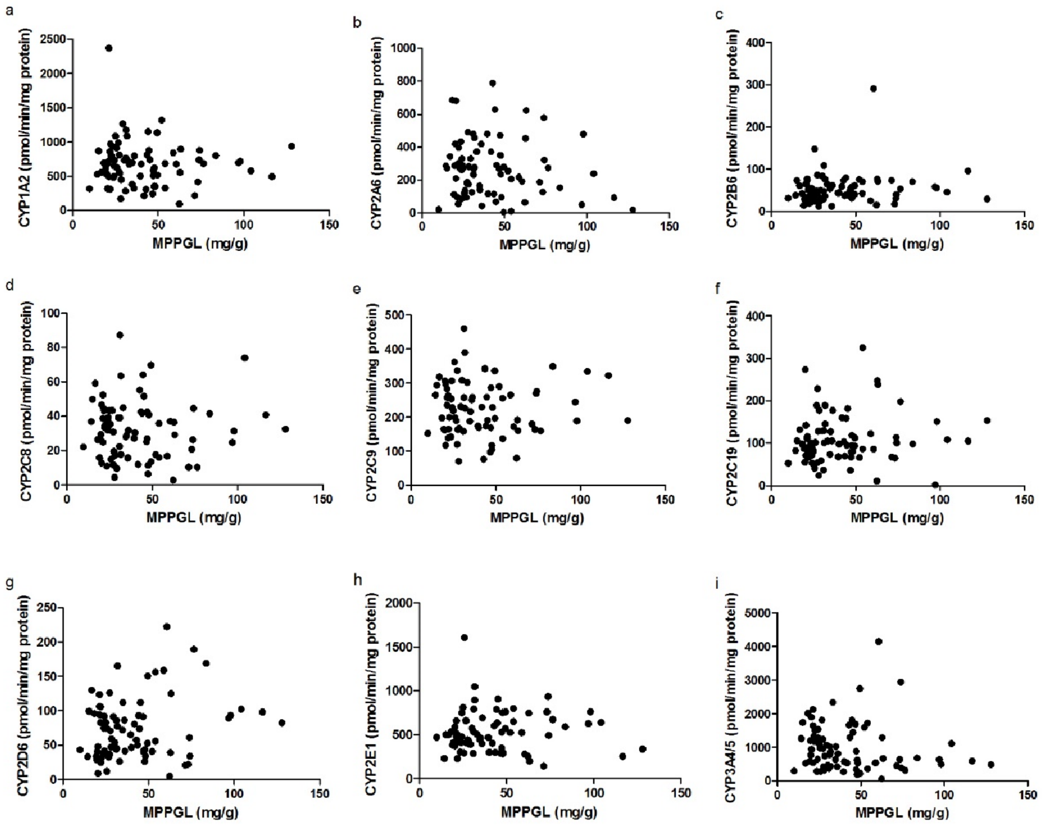

Supplementary Fig. S1. Relationship between MPPGL content and 10 CYP activity based on microsomes (V_M). There was no significant correlation between MPPGL contents and V_M of 10 CYPs (P>0.05).

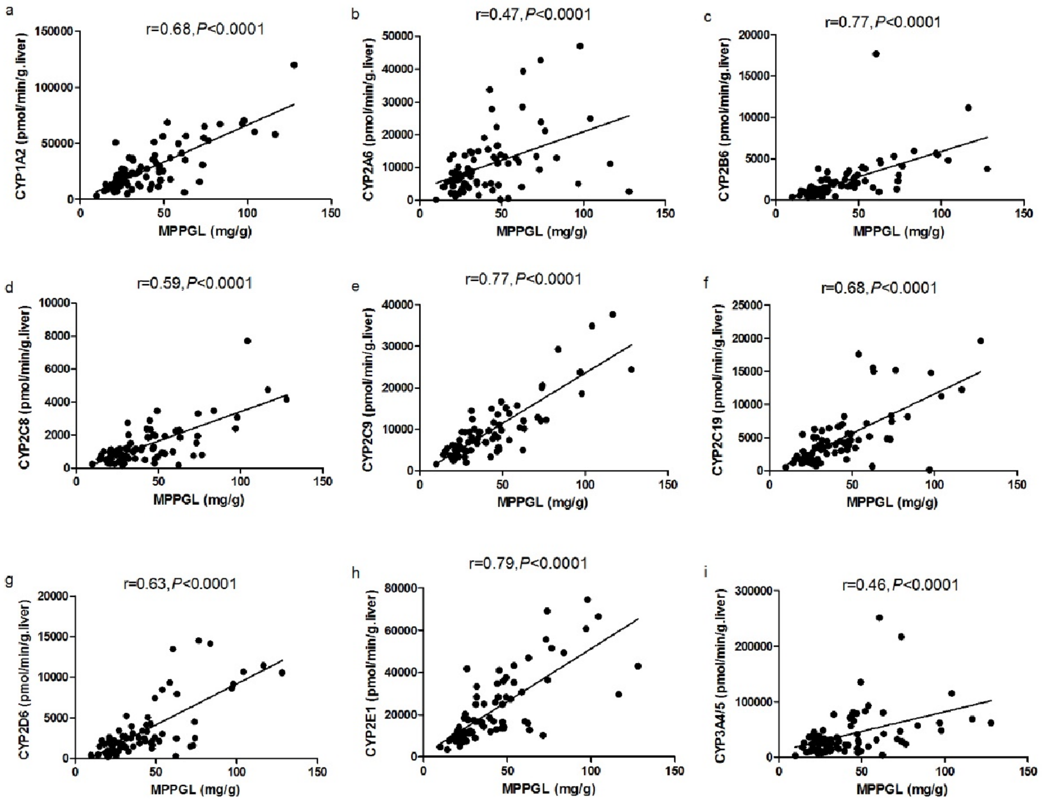

Supplementary Fig. S2. Relationship between MPPGL content and 10 CYP activity based on liver tissues (V_L). There were strong correlations ($r \geq 0.6$, $P<0.001$) between MPPGL contents and V_L of CYPs besides CYP2A6, CYP3A4/5, CYP2C8 (moderate correlations, $0.3 \leqslant r<0.6$, $P<0.001$).

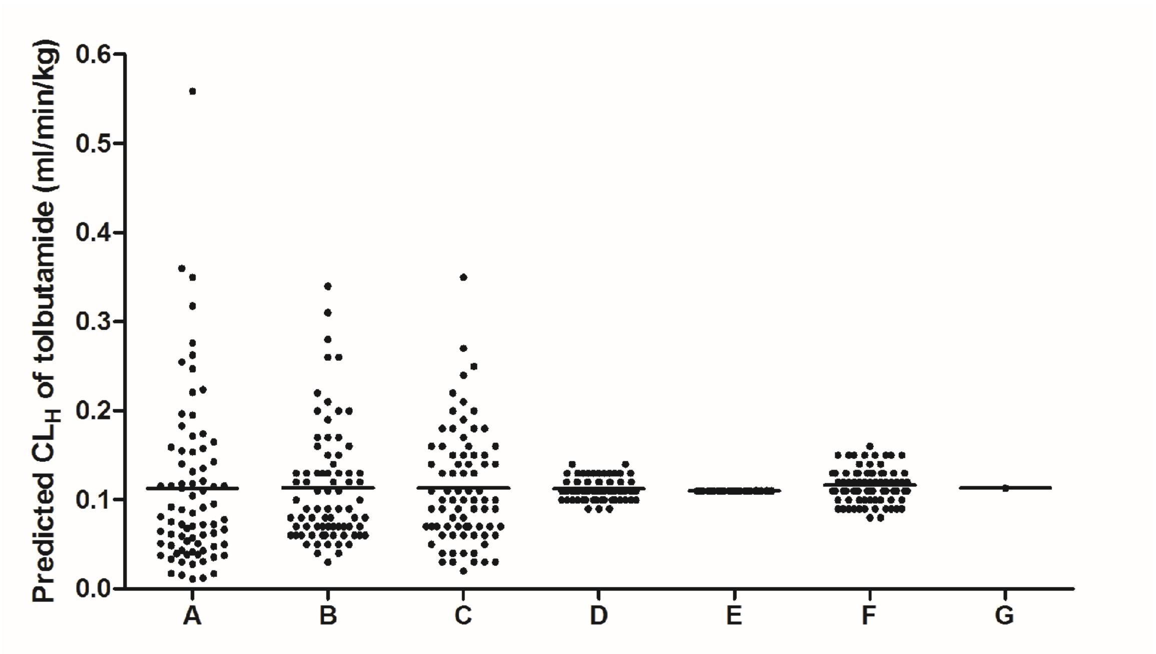

Supplementary Fig. S3. Effects of individual values for the five parameters on the prediction of hepatic tolbutamide clearance in a population of 78 samples. A to G represent the seven methods used. Method A used individual values for each parameter (MPPGL, $CL_{int,\ in\ vitro}$, LW, Q_H and BW) for 78 livers. Method B considered the individual MPPGL value and the mean values for the remaining four parameters. Similar to method B, methods C, D, E and F used the individual $CL_{int,\ in\ vitro}$, LW, Q_H or BW value, respectively, and the mean value for the remaining four parameters. Method G used mean values for the five parameters for that particular liver.
